# Therapeutic effect of hydroxyurea in treating refractory keloids

**DOI:** 10.1016/j.jdcr.2024.10.032

**Published:** 2024-11-23

**Authors:** Mayuri Vaish, Edward Hadeler, Donald A. Glass

**Affiliations:** Department of Dermatology, UT Southwestern Medical Center, Dallas, Texas

**Keywords:** hydroxyurea, keloids

## Introduction

Keloids are benign, dermal fibroproliferative growths related to skin trauma or inflammation that considerably impair physical, mental, and emotional wellbeing and quality of life.^1^ Although several options have shown success in treating keloids, there are presently no standardized guidelines for keloid management endorsed by a governing academic body.^2^ Additionally, a paucity of randomized-controlled trials makes optimizing treatment for keloids difficult. This case report describes for the first time a patient who did not respond to pentoxifylline but responded well to hydroxyurea treatment, implicating hydroxyurea as an alternative second-line systemic treatment option for refractory keloids.

## Case

A 67-year-old male with a past medical history of hyperlipidemia and no personal or family history of skin cancer presented with a chief complaint of keloids on his chest, back, shoulders, and abdomen since his late 20s. The lesions were both pruritic and painful. He denied fever, chills, and weight loss. His complete review of systems was negative except as described above. The patient had been previously treated with intralesional Kenalog injections (ILK) and surgery for some of his keloid lesions, which showed recurrence. He also underwent surgery including tissue expanders on the central chest, but that scar grew into a widespread keloid. Physical examination revealed significant hyperpigmented raised dermal plaques consistent with keloids on his shoulders, back, and lower abdomen in the waistband and groin area, as well as a large nummular-shaped white atrophic scar on the central chest with raised hyperpigmented dermal plaques on the periphery.

The patient was initially treated with intralesional 5-FU/TAC (9 parts 5-fluorouracil 50 mg/mL:1 part Kenalog 40 mg/mL) and with pentoxifylline 400 mg by mouth (PO) three times daily with meals, which was increased to 800 mg three times daily with meals after 2 months. Pentoxifylline was prescribed given its use in the prevention of keloid growth and utility in preventing keloid recurrence after excision.^3^^,^^4^ Five months after initial presentation, the patient underwent shave removal of symptomatic keloids on his left flank, left lower quadrant, and left inguinal region, with adjuvant therapy of ILK injections every 5 weeks for at least 6 months and rosiglitazone 4 mg PO daily, which he tolerated well. Despite ILK, pentoxifylline and rosiglitazone, the excised keloids regrew.

Approximately 1 year after presentation, the patient underwent shave removal of his right flank and lower abdomen. He then started methotrexate 10 mg orally weekly with folic acid 1 mg orally the other 6 days of the week. Six months later, due to lack of efficacy, the patient was switched from methotrexate to mycophenolate mofetil 1000 mg twice daily. This did not appear to help his condition despite 3 months of treatment. The patient was then switched from mycophenolate mofetil to hydroxyurea 1000 mg orally daily, which was increased to 2000 mg orally daily after 2 months.

**Fig 1:**
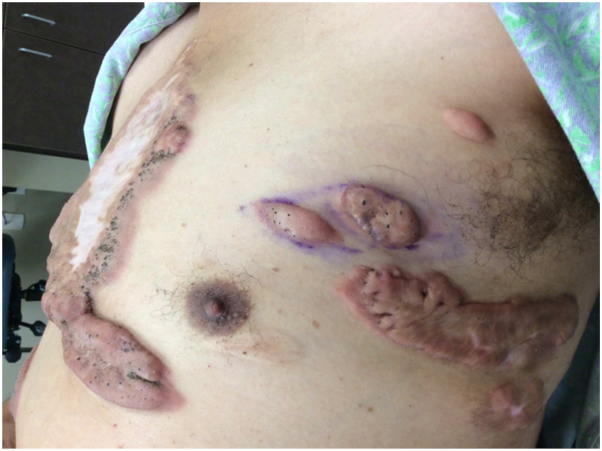
Multiple keloid lesions on the *left upper* and *lower* chest.

**Fig 2:**
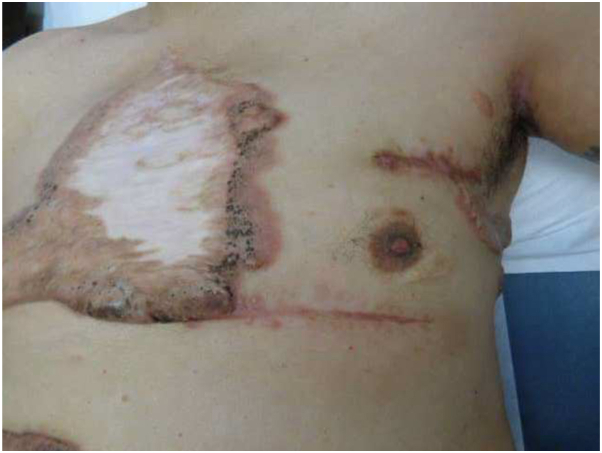
Surgical scars 4 months after excision while on hydroxyurea.

Two months after starting the 2000 mg dose hydroxyurea, the patient noted less pain and no new lesions, as well as decreased itching and discomfort. He had 2 keloids excised from the left upper chest; a month later, a larger keloid from his left lower chest was excised (Fig 1). These lesions remained stable 4 months thereafter while on hydroxyurea (Fig 2). The patient temporarily discontinued hydroxyurea and take folic acid supplementation (1 mg orally daily) for 4 months due to low-normal absolute neutrophil count. He then resumed hydroxyurea at 1500 mg orally daily and continued to not have regrowth of the excised areas and to not see any new lesions.

After approximately 3 months of hydroxyurea 1500 mg orally daily, the patient could not continue the medication due to his work and issues with insurance. Stopping the hydroxyurea led to regrowth of some of his scars and worsening of discomfort and itch.

## Discussion

This case report suggests a potential new line of research into hydroxyurea as an alternative treatment for keloids as well. Specifically, our patient experienced cessation of keloid development and progression, as well as improved itch and pain. Hydroxyurea is a DNA synthesis inhibitor not previously used in keloid management. The adult dose starts at 15 mg/kg/day and is titrated up to 35 mg/kg/day. Common side effects include constipation, headache, eczema, oral ulcers, and macrocytosis. Due to risk of bone marrow suppression, vasculitic leg ulcers, secondary leukemia or skin carcinoma, hepatotoxicity, and fetal toxicity, hydroxyurea is contraindicated in patients with bone marrow depression (WBC <2500 or platelets <100,000), severe anemia, leg ulcer wounds, HIV, and patients who are pregnant.

Hydroxyurea was chosen as a therapeutic option due to its antiproliferative properties, its previous use in treating psoriasis, and its side effect profile including “poor wound healing.” Due to this side effect, it was postulated that the medication could offset the excessive wound healing found with keloids. Also, hydroxyurea is used to treat sickle cell disease, in part due to its ability to increase the transcription of fetal hemoglobin.^5^ It is unclear whether hydroxyurea may lead to a similar conversion of skin toward its fetal state, which is less likely to scar than postnatal skin.^6^ There have been recent reports on the use of dupilumab, an interleukin-4-R inhibitor, showing promise in reducing pain, itch, and size of keloids.^7^ Hydroxyurea could therefore be used as an adjunct or instead of dupilumab in treating keloids.

The patient’s symptoms only improved with hydroxyurea, despite trying several keloid treatments including surgery, ILK, 5-fluorouracil, rosiglitazone, methotrexate, and mycophenolate mofetil. Moreover, the patient did not respond to pentoxifylline, which has shown effectiveness in treating keloids.^3^^,^^4^ Notably, the patient’s symptoms that were controlled while on hydroxyurea began to worsen upon stopping of the agent, suggesting its potency in treating keloids for this patient.

Hydroxyurea is an inhibitor of ribonucleotide reductase, a crucial enzyme to generate the deoxyribonucleotides necessary for DNA synthesis.^8^ Consequently, it induces replication stress and arrests cells in the S-phase of the cell cycle. It is possible that hydroxyurea’s unique effects on keloid treatment arise from evidence suggesting the quasi-cancerous nature of keloids such as progressive uncontrolled growth, high rates of recurrence, and lack of spontaneous regression.^9^ Particularly, keloid growths have shown the upregulation of tumor growth factor-beta/Smad and MAPK/ERK pathways as well as proto-oncogenes such as *c-jun and c-fos.*^10^ Given a potential quasi-cancerous fibroproliferative mechanism of keloids, it could be theorized that hydroxyurea halts the G0-G1 transition by preventing the formation of new DNA precursor molecules, inducing keloid fibroblast apoptosis. However, further research will be required to elucidate hydroxyurea’s mechanism on keloid scar inhibition.

## Conflicts of interest

None disclosed.
